# Rational Development of Liposomal Hydrogels: A Strategy for Topical Vaginal Antiretroviral Drug Delivery in the Context of HIV Prevention

**DOI:** 10.3390/pharmaceutics11090485

**Published:** 2019-09-18

**Authors:** Maria J. Faria, Raul Machado, Artur Ribeiro, Hugo Gonçalves, Maria Elisabete C. D. Real Oliveira, Teresa Viseu, José das Neves, Marlene Lúcio

**Affiliations:** 1 CF-UM-UP—Centro de Física das Universidades do Minho e Porto, Departamento de Física da Universidade do Minho, 4710-057 Braga, Portugaltviseu@fisica.uminho.pt (T.V.); 2 CBMA—Centro de Biologia Molecular e Ambiental, Departamento de Biologia, Universidade do Minho, 4710-057 Braga, Portugal; raulmachado@bio.uminho.pt; 3 IB-S—Institute of Science and Innovation for Bio-Sustainability, Universidade do Minho, 4710-057 Braga, Portugal; 4 CEB—Centro de Engenharia Biológica, Universidade do Minho, 4710-057 Braga, Portugal; arturibeiro@ceb.uminho.pt; 5 Paralab, SA, 4420-392 Valbom, Portugal; hugo.goncalves@paralab.pt; 6 i3S—Instituto de Investigação e Inovação em Saúde, Universidade do Porto, 4200-135 Porto, Portugal; 7 INEB—Instituto de Engenharia Biomédica, Universidade do Porto, 4200-135 Porto, Portugal; 8 CESPU, Instituto de Investigação e Formação Avançada em Ciências e Tecnologias da Saúde, 4585-116 Gandra, Portugal

**Keywords:** drug release, emtricitabine, hydrogels, liposomes, microbicides, nanomedicine, tenofovir disoproxil fumarate, topical PrEP

## Abstract

HIV/AIDS stands as a global burden, and vaginal microbicides constitute a promising strategy for topical pre-exposure prophylaxis. Preceding the development of a microbicide containing tenofovir disoproxil fumarate (TDF) and emtricitabine (FTC), in silico and in vitro studies were performed to evaluate the physicochemical characteristics of both drugs, and to study their biophysical impact in lipid model systems. Results from these pre-formulation studies defined hydrogels as adequate vehicles to incorporate TDF-loaded liposomes and FTC. After studying interactions with mucin, zwitterionic liposomes with a mean diameter of 134 ± 13 nm, an encapsulation TDF efficiency of approximately 84%, and a transition temperature of 41 °C were selected. The chosen liposomal formulation was non-cytotoxic to HEC-1-A and CaSki cells, and was able to favor TDF permeation across polysulfone membranes (*J*_ss_ = 9.9 μg·cm^−2^·h^−1^). After the incorporation of TDF-loaded liposomes and FTC in carbomer hydrogels, the drug release profile was sustained over time, reaching around 60% for both drugs within 3–6 h, and best fitting the Weibull model. Moreover, liposomal hydrogels featured pseudoplastic profiles that were deemed suitable for topical application. Overall, the proposed liposomal hydrogels may constitute a promising formulation for the vaginal co-delivery of TDF/FTC.

## 1. Introduction

Since the beginning of the epidemic, HIV has infected more than 77 million people and caused around 35 million deaths [1]. By 2017, the Joint United Nations Program on HIV/AIDS (UNAIDS) estimated that 36.9 million people worldwide were infected with the virus, and that the female population accounted for more than half of these infections. In addition, sexual transmission remains the leading cause of new infections [2]. Although considerable progress has been made since antiretroviral (ARV)-based therapies were introduced, HIV/AIDS stands as an increasing global health concern, and new prevention strategies to prematurely fight and control the virus dissemination are needed [3]. Recently, ARVs have been explored for oral and topical pre-exposure prophylaxis (PrEP) in HIV-negative individuals before exposure to the virus [4].

Oral PrEP with tenofovir-based regimens is currently available in many countries worldwide and is already considered a well-established preventive strategy [5]. The once daily administration of a combination of 300 mg of tenofovir disoproxil fumarate (TDF) and 200 mg of emtricitabine (FTC), available commercially as Truvada^®^ (Gilead, Foster City, CA, USA), has been the most commonly used regimen. Conversely, no topical PrEP product is available. Apart from efficacy issues, the preference for oral PrEP is highly correlated with the fact that oral administration is convenient, discreet, and relatively affordable [6]. However, low patient adherence to daily regimens and ARVs poor pharmacokinetic profiles and side effects may still compromise the success of oral PrEP and decrease efficacy [5]. Also, the need for the persistent daily intake of ARVs may be regarded by some populations as cumbersome, making on-demand products, i.e., requiring administration/application around the time of sexual intercourse, more attractive [7]. Topical microbicides, particularly those of vaginal use, arose as an interesting approach for topical PrEP in alternative to the oral regimens, and their protective effect in women, although modest, was indeed observed in previous clinical trials for a tenofovir gel [8] and a dapivirine ring [9,10]. Vaginal microbicides are intended for preventing male-to-female transmission of the virus and act at the early stages of infection, namely at the cervicovaginal mucosa. The relatively high local ARV drug concentrations—which can be rapidly achieved upon vaginal administration of a microbicide—and negligible systemic exposure are regarded as advantageous features when comparing to oral PrEP [4]. Despite all mentioned advantages, vaginal drug delivery is challenging. Various formulation and delivery approaches have been tested, ranging from more ‘traditional’ drug dosage forms (e.g., vaginal tablets, gels, and creams) to more ‘advanced’ systems (e.g., vaginal rings). The development of nanotechnology-based systems, in particular, is thought to hold the potential to improve the prophylactic performance of ARV drugs by providing their sustained release and sustaining protective concentrations at the mucosa [11]. Furthermore, the possibility of modulating specific drug targeting through, for example, functionalization of the nanosystem surface, is also possible [12].

Lipid-based nanocarriers have been extensively explored for vaginal drug delivery for the treatment of fungal and bacterial diseases [13,14,15], as well as viral infections by human papillomavirus (HPV) [16] and herpes simplex virus type 2 (HSV-2) [17]. However, the application of lipid formulations in HIV prophylaxis is sparse, and few research groups investigated their potential to act as nanomicrobicides for the vaginal delivery of ARVs. Among the first reports is the study from Caron et al. [18] in rhesus macaques infected with a recombinant simian immunodeficiency virus containing the RT coding region from HIV-1 (RT-SHIV), in which liposomes loaded with the nonnucleoside reverse transcriptase inhibitor (NNRTI) MC1220 were developed, and further included in a carbomer hydroxypropyl cellulose and glycerol mixture gel for vaginal administration. Vaginal administration was made with a single application of liposomal gels with 0.5% and 1.5% of MC1220, or by applying a 0.5% gel for four days. Intermediate protection against SHIV challenge was observed with all the administered formulations. However, the higher concentration of MC1220 failed to increase the protection levels, which was probably related with MC1220 low solubility. In another study, Alukda et al. [19] prepared solid lipid nanoparticles functionalized with polylysine–heparin for the vaginal delivery of tenofovir. A low drug loading was obtained (~0.1%), which was probably due to tenofovir hydrophilic character. Additionally, the formulation did not exhibit in vitro cytotoxicity up to concentrations of 0.9 mg·mL^−1^. Overall, solid lipid nanoparticles demonstrate interesting properties, but further studies with more lipophilic or hydrophobic drugs were deemed necessary. Furthermore, Malavia et al. [20] assessed the antiviral activity of different plain liposomes composed with naturally occurring and synthetic lipids. Even if the intrinsic activity of liposomes against HIV-1 was relatively low, at least to be considered as single active agents, this study indicates that selected formulations may be interesting carriers for use in vaginal PrEP approaches.

All in all, available studies have shown the potential to invest in lipid formulations for topical HIV PrEP. Also, the success of Truvada^®^ in oral PrEP seems to indicate that TDF/FTC may be an interesting drug combination for advancing this particular delivery strategy [21]. Previous reports on animal and human studies of vaginal rings containing TDF/FTC seem to support that the combination is useful for topical PrEP [22,23,24]. Thus, the aim of the current work is the development of a putative topical microbicide based on a liposomal hydrogel containing TDF/FTC. A preliminary study conducted to evaluate the interaction of these drugs with lipid membrane models highlighted their distinct needs in terms of carriers. TDF was shown to be well distributed in lipid bilayers, while causing no disturbance regarding the biophysical stability of liposomes. Contrastingly, FTC was shown able to yield strong biophysical impairment at the polar headgroups of lipid bilayers, which indicated that liposomes were not adequate carriers for this drug. Therefore, the final formulation consisted of liposomes loaded with TDF and further incorporated in carbomer-based hydrogels with FTC. Such a final dosage form was chosen due to its good acceptability by women, technological versatility, and low manufacturing cost, although some limitations, in particular messiness or leakage, are well recognized [25]. These last problems can usually be minimized by simply using lower volumes of gel per administration.

To the best of our knowledge, this is the first study proposing the combination of lipid nanocarriers and hydrogels for the intravaginal co-delivery of TDF and FTC. Particular to this work are the pre-formulation and formulation studies that allowed a better understanding of the physicochemical properties of the considered drugs under vaginal conditions and, consequently, planning of the rational development of the formulation in order to select the most suitable vehicles to deliver TDF and FTC.

## 2. Materials and Methods

### 2.1. Materials

Zwitterionic—1,2-dioleoyl-*sn*-glycero-3-phosphocholine (DOPC), 1,2-dimyristoyl-*sn*-glycero-3-phosphocholine (DMPC), 1,2-dipalmitoyl-*sn*-glycero-3-phosphocholine (DPPC), 1,2-distearoyl-*sn*-glycero-3-phosphocholinephosphatidylcholine (DSPC), cationic—dioctadecyldimethylammonium bromide (DODAB), and anionic lipids—1,2-dimyristoyl-*sn*-glycero-3-phosphorylglycerol (DMPG), as well as cholesterol (CHOL) were obtained from Avanti Polar Lipids Inc. (INstruchemie, Delfzyl, The Netherlands). TDF, a prodrug of the nucleotide reverse transcriptase inhibitor (NtRTI) tenofovir, was purchased from Kemprotec (Cumbria, UK), and the nucleoside reverse transcriptase inhibitor (NRTI) FTC was purchased from Sequoia Research Products (Pangbourne, UK). Carbomer 2001 (PFC^®^) was provided by Guinama Laboratories (Valencia, Spain). Purified type II mucin from porcine stomach and thiazolyl blue tetrazolium bromide (MTT) were acquired from Sigma-Aldrich (St. Louis, MO, USA). All other materials and solvents were provided by Merck (Kenilworth, NJ, USA) and were of analytical grade or equivalent.

### 2.2. In Silico Studies

TDF and FTC biopharmaceutical properties are relevant information when selecting the most suitable liposomal vehicle. To this end, in silico pre-formulation studies were carried out using the Chemaxon^®^ software (ChemAxon, Budapest, Hungary) integrated with the MarvinSketch^®^ mode (ChemAxon, Budapest, Hungary). The chemical structure of both drugs was introduced, and chemical descriptors such as size, geometry, lipophilicity, ionization, solubility, and surface topology were determined.

### 2.3. Preparation of Liposomes and Liposomal Hydrogels

Liposomes were prepared by the classical thin film hydration method [26,27,28]. Briefly, lipids were dissolved in chloroform, and the organic solvent was evaporated at 37 °C under a constant nitrogen stream to obtain a dry lipid film. The film was further hydrated with ultrapure water, above the lipid main phase transition temperature. Afterwards, the lipid suspension was vortexed to yield multilamellar vesicles (MLVs) with a final concentration of 4 mM. Large unilamellar vesicles (LUVs) were then prepared by extrusion (10 passages) of the MLVs suspension through polycarbonate filters with a pore size of 100 nm (Millipore SAS, Molsheim, France). Extrusion was also processed at a temperature above the lipid main phase transition temperature (Table 1).

For mucoadhesion studies, liposomes with different surface charges: zwitterionic (composed by DMPC), anionic (composed by DMPG), and cationic (composed by DODAB) were prepared according to the classical thin film hydration method, followed by extrusion as described above.

For TDF encapsulation, zwitterionic liposomes were developed by using different lipids sharing the same zwitterionic headgroup region (phosphatidylcholine) but comprising different saturated fatty acids, namely myristic acid (CH_3_(CH_2_)_12_COOH), palmitic acid (CH_3_(CH_2_)_14_COOH), stearic acid (CH_3_(CH_2_)_16_COOH), or the unsaturated oleic acid (CH_3_(CH_2_)_7_CH=CH(CH_2_)_7_COOH). Formulations of each lipid composition (DMPC, DPPC, DSPC, and DOPC) were also prepared without cholesterol and with 30% or 40% cholesterol. Table 1 presents a summary of all the liposomes prepared and the experimental conditions used.

To determine the most effective encapsulation procedure, TDF-loaded zwitterionic liposomes were prepared using three different methodologies, all conducting to a final TDF concentration of 40 μM: (i) thin film hydration, (ii) incubation, and (iii) direct mixing. In the first, the lipid film was prepared as described above, and hydration of the lipid film was achieved by the addition of an aqueous solution of TDF above the lipid main phase transition temperature. In the case of incubation method, TDF was added to LUVs that were prepared as described previously. The encapsulation of TDF by the direct mixing method was conducted by co-drying the lipid organic solution with an ethanolic drug solution. Then, the resulting lipid–drug film was hydrated, and liposomes were prepared as mentioned above. A schematic representation of the encapsulation methods is presented in Supplementary Materials (Figure S1).

Carbomer 2001 (0.5% *w*/*w*) was used as a gelling agent in order to prepare hydrogels for vaginal administration. Briefly, the polymer was dispersed in ultrapure water under constant stirring, being further neutralized with triethanolamine (≈0.13% *w*/*w*) to promote gelation. Liposomes containing TDF were directly included into the gel by replacing part of the external phase with the aqueous dispersion of lipid colloidal carriers (10% *w*/*w*). To prepare FTC-loaded hydrogels, the drug was first dissolved in ultrapure water in order to obtain a final concentration of 7.19 × 10^−4^ M. Then, the polymer was added to the external phase, being further neutralized with triethanolamine to promote gelation.

### 2.4. Small-Angle and Wide-Angle X-ray Scattering (SAXS/WAXS)

To assess the order and packing of lipid bilayers, X-ray measurements were conducted at the Austrian SAXS/WAXS beamline at the synchrotron light source ELETTRA (Trieste, Italy) employing a monochromatic synchrotron radiation with wavelength of 0.15 nm.

The studied samples consisted of non-extruded DPPC lipid suspensions without the drugs, with TDF, with FTC, and TDF:FTC (3:2 *w*/*w*). The drugs were co-solubilized with DPPC in a mixture of chloroform/methanol (9:1 *v*/*v*) to obtain the desired drug:lipid molar fraction (1:9). The final lipid concentration in each sample was approximately 15–20% *w*/*v*. After the total dissolution of the compounds, the lipid suspensions were prepared as previously described [29] and later transferred into X-ray transparent glass capillaries with 1.5-mm diameter (Hilgenberg, Malsfeld, Germany), which were flame-sealed and stored at 4 °C until measurement.

*P*-bromo benzoic acid (WAXS) and silver behenate (SAXS) patterns were recorded using a Pilatus 100K 2D and Pilatus 1M detector system, respectively, with a pixel size of 172 μm at positions that covered the typical diffraction spacing range (s = 2sinθ/λ, where λ is the wavelength and 2θ is the scattering angle) of interest. The diffraction spacings were calibrated using the lamellar peaks of silver behenate (SAXS) and *p*-bromo benzoic acid (WAXS) as standards. Static exposures were taken below and above the main transition temperature as controlled by a thermostated water bath (stability ± 0.1 °C; Unistat CC, Huber, Offenburg, Germany) in order to obtain the diffraction patterns typical of DPPC lipid phases (L_β′_ and L_α_) and evaluate the effect of drugs in such phases. Data analysis was made as described elsewhere [30].

### 2.5. Attenuated Total Reflection–Fourier Transform Infrared (ATR-FTIR)

The effect of TDF and FTC in the microviscosity of lipid formulations was evaluated by ATR-FTIR to characterize the vibrational frequencies of chemical groups representative of the main phospholipid regions (hydrophilic head, hydrophobic fatty acid tail, and an interfacial area between head/tail). Briefly, 50 μL of each sample was added to crucibles made of aluminum foil, and the aqueous solvent was evaporated in order to form a lipid film. The measurements were conducted by pressing the lipid film against the crystal from the ATR accessory (refraction index = 2.4; penetration depth = 1.66 μm) in a Spectrum Two Spectrometer (Perkin-Elmer, Waltham, MA, USA). Spectra were recorded in the spectral range of 400–4000 cm^−1^, with increments of 4 cm^−1^ and 16 scans per spectrum.

### 2.6. Dynamic Light Scattering (DLS) Applied to Microviscosity Studies

DLS measurements using a ZetaSizer Nano ZS (Malvern Panalytical, Malvern, UK) were carried out to evaluate the effect of TDF (40 μM) in the main phase transition temperature (*T*_m_) and cooperativity (B) of DPPC liposomes (4 mM), as previously reported [30,31].

The samples were heated from 25 °C to 55 °C, with intervals of 0.5 °C. Measurements were made at each temperature with a previous stabilization time of 60 s. The data obtained was expressed as mean count rate (MCR) versus temperature (T), and the results were fitted following a modified Boltzmann equation:

(1) $$MCR=m_{1}+b_{1}T+\frac{m_{2}-m_{1}+b_{2}T-b_{1}T}{1+10^{B\;\left(\frac{1}{T}-\frac{1}{T_{m}}\right)}}$$

where *m*_1_ and *m*_2_ are the slopes of the linear fits to the data before and after the phase transition region, respectively, and *b*_1_ and *b*_2_ are the corresponding y-axis intercepts.

### 2.7. Mucoadhesion Studies

In order to assess the mucoadhesive properties, liposomes (400 μM) with different surface charges (positive, negative, and neutral) (Table 1) were evaluated regarding their size (diameter), polydispersity index, and zeta potential before and after incubation with a mucin suspension (0.4 mg·mL^−1^). The mean hydrodynamic diameter (Z-average) and polydispersity index (PDI) were measured by DLS using a ZetaSizer Nano ZS at a fixed backscatter angle of 173°. Z-average and PDI values were obtained from the correlogram by the ZetaSizer Nano ZS software (Malvern Panalytical, Malvern, UK) after cumulative analysis. Zeta potential was measured by electrophoretic light scattering (ELS). In this case, a Dip cell^®^ (Malvern Panalytical) was introduced in polystyrene cells, and the zeta potential values were calculated from the conversion of electrophoretic mobility according to the method of Helmholtz–von Smoluchowski [32].

Furthermore, the quenching effect of the different charged systems was evaluated on the intrinsic fluorescence of mucin. The fluorescence measurements were carried out on a Perkin-Elmer LS 50B fluorimeter, using an excitation wavelength of 270 nm and an emission range of 300 to 500 nm.

### 2.8. Encapsulation Efficiency

The encapsulation efficiency (EE%) was determined using centrifugal filter devices with a MW cut-off of 100 kDa (Amicon^®^ Ultra, Millipore, Burlington, MA, USA). The EE% of TDF was calculated by the difference between the total amount of drug used to prepare the formulation and the amount of free drug remaining in the aqueous phase. Briefly, one milliliter of the lipid formulation containing the drug was transferred to the filter units, which were further centrifuged in a Hettich Universal 320 centrifuge (Vlotho, Germany) at 3000 rpm for approximately 20 min. The unentrapped drug was able to diffuse into the filtrate, and its content was quantified using a UV-2501 PC spectrophotometer (Shimadzu, Kyoto, Japan) at 200–400 nm. The EE% was determined using the following equation:

(2) $$EE\%=\frac{{\left[TDF\right]}_{total}-{\left[TDF\right]}_{free}}{{\left[TDF\right]}_{total}}\times 100$$

The amount of TDF was quantified at a wavelength of 260 nm (Ɛ_TDF_ = 13570 M^−1^·cm^−1^) by a validated UV/Vis spectrophotometry method according to the International Conference on Harmonization (ICH) Q2(R2) guideline using a Shimadzu UV-2501 PC spectrophotometer.

### 2.9. Liposomal Size, Polydispersity Index, and Zeta Potential

The Z-average and PDI of liposomes (free or loaded with TDF) and zeta potential were determined by DLS and ELS, as described in Section 2.7. Before measurements, liposomal samples were diluted in ultrapure water (3:7 *v*/*v*) to attain suitable scattering intensity. All measurements were performed at 25 °C to mimic shelf-stability conditions.

### 2.10. In Vitro Drug Release Studies

In vitro studies were performed in order to evaluate TDF and FTC release profiles from their final dosage form following the dialysis bag (SnakeSkin 3500 Da MWCO, Thermo Fisher Scientific, Eindhoven, The Netherlands) diffusion method. The dosage forms tested were the hydrogel containing FTC (designated as FTC@HG) and the hydrogel containing liposomes loaded with TDF (designated as (DPPC+TDF) @HG).

Briefly, 2 mL of each dosage form was transferred to dialysis bags; afterwards, they were immersed in 15 mL of ultrapure water, which was used as a receptor medium. The receiving phase was incubated in a thermostatically controlled shaking (120 rpm) water bath at 37 °C. At pre-determined time intervals, one milliliter of the receptor medium was removed and replaced with the same volume of fresh medium to maintain sink conditions. The amount of TDF or FTC released from the dialysis bag was quantified at wavelengths of 260 and 280 nm, respectively (Ɛ_TDF_ = 13,570 M^−1^·cm^−1^ and Ɛ_FTC_ = 9160 M^−1^·cm^−1^), by a validated UV/Vis spectrophotometry method according to the International Conference on Harmonization (ICH) Q2(R2) guideline using a Shimadzu UV-2501 PC spectrophotometer (Shimadzu, Kyoto, Japan).

The studies were conducted over 7 h (considered as representative of the time period around sexual intercourse), and results were presented as the cumulative percentage of released compound versus time. The in vitro release profiles were fit with different mathematical models (GraphPad Prism^®^ software, San Diego, CA, USA) to evaluate the release kinetic profile and elucidate about the mechanisms that control drug release [33]. Considered models were: first-order, Higuchi, Korsmeyer–Peppas, and Weibull. The model with the highest value of adjusted coefficient of determination was selected as the best fit to the drug release profile.

### 2.11. In Vitro Permeation Studies

A comparison between the in vitro permeation of non-encapsulated drug (designated as TDF) and encapsulated TDF (designated as DPPC+TDF) was carried out in a Franz Cell system (V-Series Stirrers, PermeGear, Hellertown, PA, USA) with a diffusion area of 0.64 cm^2^.

Briefly, the system was maintained at a constant temperature of 37 °C using a water bath. Samples (800 μL) were transferred to the acceptor compartment, and a predefined volume of ultrapure water (5 mL) was used as receptor medium. The drug permeation rate was measured using a synthetic polysulfone membrane placed between the two compartments. At pre-determined time intervals (0, 1, 2, 4, 6, 8, 24 h), aliquots of 400 μL of the receptor medium were removed and replaced with the same volume of fresh medium to maintain sink conditions. The amount of drug that permeated through the membrane was quantified using a UV-2501 PC spectrophotometer at 200–400 nm, as described in Section 2.8.

Permeation parameters were interpreted from the cumulative amount of released drug per unit membrane area (Q_R_/A) versus time (t) plot. The gradient and *x* intercept of the linear portion of the plot yielded steady-state flux (*J*_ss_) and lag time (*t*_L_), respectively. *J*_ss_ (μg·cm^−2^·h^−1^) was calculated by the following equation:

(3) $$J_{SS}=\frac{Q_{R}}{At}$$

where *t* (h) is permeation time, *A* (cm^2^) is permeation area, and Q_R_ (μg) is permeated amount of drug. The drug permeability *K_p_* (cm·h^−1^) was calculated by the following equation:

(4) $$K_{P}=\frac{J_{SS}}{C_{d}}$$

where *C*_d_ is the initial concentration in the donor chamber (μg·cm^−3^).

### 2.12. Rheological Behavior of Hydrogels

After mechanical and thermal equilibration, hydrogels (designated as HG) and liposomal hydrogels (empty: DPPC@HG; or loaded with both drugs: (DPPC+TDF)/FTC@HG) were sheared in an EVO Expert L series Rotational Viscometer (FungiLab, Hauppauge, NY, USA) at a shear rate adequate for the spindle used to obtain the flow patterns. Gel apparent viscosity and shear stress were recorded as a function of shear rate. Each hydrogel was stirred for one minute at each shear rate (0, 2, 4, 6, 8, 10, 12 rpm), and the corresponding forward and backward rheograms were recorded, thus allowing the examination of flow curves and the possible existence of hysteresis loops.

### 2.13. Cytotoxicity Studies

The in vitro toxicity of drugs (FTC and TDF) and liposomes (unloaded: DPPC or DODAB or loaded with TDF: DPPC+TDF or DODAB+TDF) to human cervical CaSki and endometrial HEC-1-A cell lines (ATCC, Manassas, VA, USA) was assessed using the MTT metabolic viability assay. CaSki cells were maintained in RPMI 1640 medium (Invitrogen, Carlsbad, CA, USA) and HEC-1-A cells in McCoy’s 5A modified medium (Invitrogen), respectively. In both cases, media was supplemented with 10% (*v*/*v*) fetal bovine serum (Merck Millipore, Burlington, MA, USA), 100 U·mL^−1^ penicillin (Merck Millipore) and 100 μg·mL^−1^ streptomycin (Merck Millipore). Cells were maintained under routine conditions (37 °C, 5% CO_2_, and 95% RH), while media were changed 2–3 times weekly.

Briefly, cells were initially seeded in 96-well plates (10^4^ cells/well) and left in culture for 24 h. Afterwards, cells were incubated for 24 h with drugs or liposomes (plain or drug-loaded) dispersed in media. Then, the cells were washed twice with phosphate-buffered saline and incubated for 4 h with medium containing 0.5 mg·mL^−1^ of MTT. The resultant formazan crystals were dissolved with 200 μL of dimethyl sulfoxide, and cell viability was measured using a plate reader at the absorbance of 570 nm (readings at 630 nm were used for background deduction). Plain medium and 1% (*v*/*v*) Triton X-100 in media were used as negative and positive controls, respectively.

## 3. Results

### 3.1. Pre-Formulation Studies

Pre-formulation studies are a stepping stone in the rational development of an effective formulation, and several factors should be considered during this process. It is crucial to initially evaluate and conduct a comprehensive study of the physicochemical and biopharmaceutical properties of considered drugs [34], which can be achieved by in silico approaches. In addition, understanding the formulation targets as well as the barriers they impose is of major importance and may dictate new approaches in the formulation development process, such as drug–membrane interaction studies. These are also of use in providing better knowledge of the drugs and guiding the development of formulation [29,35].

#### 3.1.1. In Silico Studies

**Figure 1:**
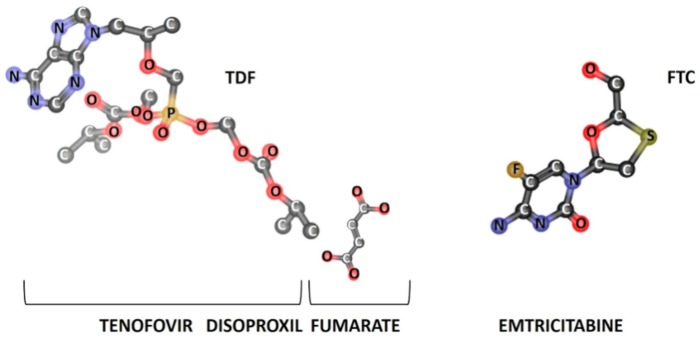
Three-dimensional (3D) chemical structures of tenofovir disoproxil fumarate (TDF) and emtricitabine (FTC). Chemical structures were generated with ‘Chemicalize’ tool from Chemaxon^®^ software.

Relevant physicochemical properties of TDF and FTC (Figure 1) were predicted in silico (Table 2) and subsequent inferences about their ionization, lipophilicity, permeability, and solubility, as well as their classification according to the Biopharmaceutical Classification System (BCS), were made.

Regarding the FTC ionization profile, the calculated pKa value (pKa = 1.75) was lower than the experimental value reported previously (pKa = 2.65) [36]. The pKa of FTC was correlated with the ionization of the primary amine functional group (Figure 1, amine bounded to the heterocycle group in FTC). However, according to the Henderson–Hasselbalch equation, the experimental pKa of 2.65 corresponds to a very low dissociation constant (Kb = 4.47 × 10^−12^). Considering the theoretical value obtained, the FTC dissociation constant will be even lower (Kb = 5.62 × 10^−13^), thus indicating that the ionization of the primary amine is unlikely to occur. This is particularly relevant when considering physiological conditions, since such low pH values are not observed at the vagina, neither pre-ejaculation (pH = 3.5−4.5) or post-ejaculation (pH = 7.0−8.5) [37]. The theoretical pKa of TDF agreed with the experimental value (pKa = 3.75) [36]. Although there may be a predominance of a positively charged TDF isoform at lower physiological pH values due to the amine group protonation (Figure 1, amine bounded to the heterocycle group in TDF), it is anticipated that TDF will be predominately in the non-ionized state at relevant pH values expected for topical vaginal administration.

According to the Lipinski “rule of five”, it is more likely for a compound to exhibit poor permeability if at least two of the following characteristics are observed: LogP >5; molecular weight (MW) >500 Da; H donors >5 and H acceptors >10 [38]. Thus, FTC fulfills the requirements for presenting good permeability, while TDF is expected to have permeability issues given its high MW and number of H acceptors groups (Table 2). Regarding lipophilicity, TDF and FTC are quite different. Moreover, FTC can be classified as hydrophilic (logP < 0), while TDF is moderate lipophilic (0 < logP < 3). However, concerning aqueous solubility, both drugs are predictably water-soluble (>1 mg·mL^-1^).

The good permeability of FTC places this compound in BCS class I, which is in agreement with official classification [39]. However, one should also consider the FTC hydrophilic nature, which may qualify it as a BCS class III drug, which is actually in agreement with a recent revision of the BCS classification [40]. TDF is only moderately lipophilic, and due to its poor permeability, it can be classified as a class III drug, which is also in agreement with its reported classification [39,40]. Thus, both drugs may benefit from formulation strategies that can improve their permeability and enhance tissue penetration, namely to the level of the epithelium and lamina propria, where most HIV-susceptible cells are located [41]. Moreover, the higher polarizability and higher affinity of TDF drug to lipidic environments (demonstrated by PSA and logP values in Table 2, respectively) may compromise its capacity to diffuse in cervicovaginal mucous fluids. Indeed, PSA values refer to the surface area of the oxygen and nitrogen atoms; thus, high PSA values suggest that TDF may be highly polarized and able to interact with mucins by hydrogen bonding [42,43]. The moderate lipophilic character of TDF can also contribute to non-specific binding to hydrophobic domains of mucins, which may further reduce its diffusion in mucus [44]. Therefore, TDF would benefit from its encapsulation in nanocarrier systems, such as liposomes, that protect the drug from interacting with mucins and potentially enhance its distribution across mucus. Liposomes have an amphiphilic character that can provide the necessary lipophilic environment for TDF encapsulation, while displaying hydrophilic surface that promotes transport in mucus. On the contrary, FTC is highly hydrophilic, thus not constituting a problem for cervicovaginal mucus transport, but can contribute to its premature leakage from the vaginal cavity. Thus, hydrogels seemed to be an interesting approach to increase FTC retention in the vagina, given the viscoelastic nature of such systems and their typical mucoadhesive behavior [25].

In summary, drug properties predicted by in silico studies helped us decide on the development of a carbomer-based hydrogel containing FTC and liposomes loaded with TDF as a potential vaginal microbicide.

#### 3.1.2. Drug–Membrane Interaction Studies

Molecules with an intracellular mechanism of action such as TDF and FTC need to bypass the membrane of HIV-susceptible cells in order to reach the cytoplasm. There, these drugs can be phosphorylated into their active forms and compete with endogenous nucleotides to terminate the formation of viral DNA [45]. In this context, their ability to interact with cellular membranes may further dictate the ability of both TDF and FTC to penetrate or even distribute in the genital tract.

Phosphatidylcholines, including the palmitoyl derivative (DPPC), are phospholipids commonly used to mimic the physicochemical and biological properties of cellular membranes [46]. Membrane model systems made of DPPC exhibit many features of the cellular membranes (e.g., the barrier and transport function, or permeability by passive diffusion through the bilayer). Drugs may alter phospholipidic membrane properties, namely its structure, lipid-packing parameters, and microviscosity [35]. In this regard, the effects of TDF and FTC in different DPPC lipid phases were evaluated by SAXS and WAXS diffraction patterns, providing information on the bilayer structure and lipid packing through the respective measurement of long and short spacing (d values). Since lipid bilayer organization, thickness, and phospholipids packing are temperature dependent, the measurements were conducted at 20 °C and 55 °C in order to assess the effect of TDF and FTC on the gel (L_β′_) and fluid (L_α_) phases of DPPC, respectively.

**Figure 2:**
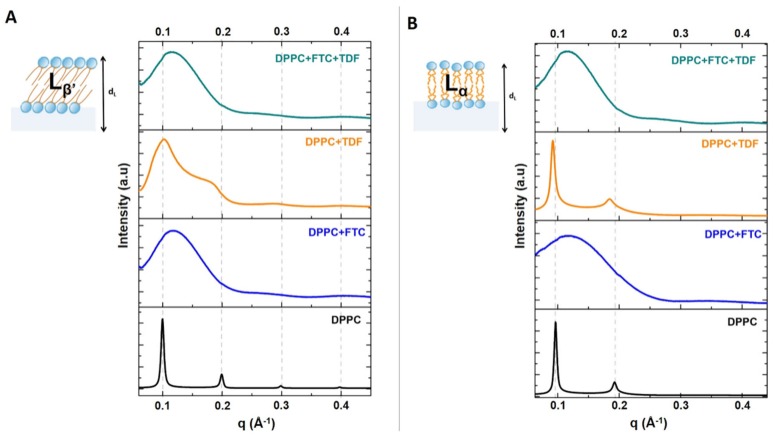
Small angle X-ray diffraction silver behenat (SAXS) patterns of DPPC (black) or DPPC containing TDF (yellow), FTC (blue), or TDF and FTC (3:2 *w*/*w*) (green) measured in the L_β_′ (**A**) and L_α_ (**B**) phases of DPPC.

Typical SAXS patterns (Figure 2) were obtained for L_β′_ and L_α_ phases of fully hydrated DPPC in good agreement with our previous reports [47]. The lamellar long spacing (bilayer thickness, including a water layer between the bilayers) was determined in each lipid phase from the SAXS patterns of DPPC, and the values are presented in Table 3.

The diffraction peaks of the DPPC membranes in the absence of the drugs presented a small full width of the peaks at one-half of their intensity (FWHM), indicating a good correlation between the bilayers (see values of correlation length ξ in Table 3). This correlation was affected by the addition of either TDF or FTC. However, the effect of each drug in the membrane structure was quite different. TDF has the least disturbing effect on membrane structure, as it only slightly increased the long-spacing value in the L_α_ phase. This event may result in modifications in DPPC hydration due to interactions with TDF. Furthermore, the ξ values in the L_α_ phase, although reduced in comparison with native DPPC layers, were still high, thus indicating that the drug insertion does not disturb the structure of the lipid bilayers (Table 3). The long-space value of DPPC in the L_β′_ phase was not notably changed upon the addition of TDF, but a larger reduction of ξ indicates a higher disturbance effect of this drug on membrane structure.

The addition of FTC to the lipid system led to a noticeable breakdown of the multilamellar correlation (decrease ξ values in comparison with DPPC without drug, as shown in Table 3), thus manifesting the greater disturbance effect of this drug on the membrane structure. In both phases, the inclusion of FTC promoted a decrease in long-spacing values, which indicates a decrease in the thickness of the lipid bilayer plus the hydration layer. This decrease might be due to a fluidizing effect of the drug in the lipid bilayer, resulting in a reduction in the bilayer thickness and/or to a decrease in the water layer.

**Figure 3:**
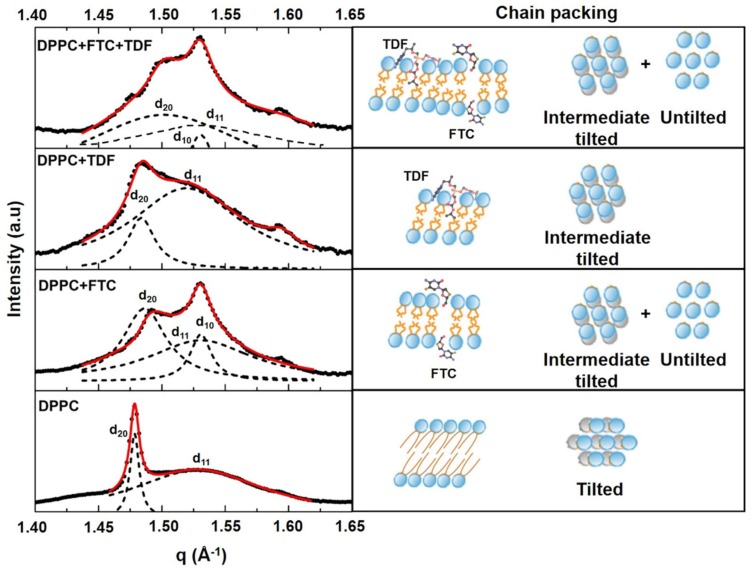
Wide angle X-ray diffraction (WAXS) patterns of DPPC or DPPC containing TDF, FTC, or TDF and FTC (3:2 *w*/*w*) measured in the L_β′_ phase of DPPC. Solid red lines give the best fit of the Lorentzian’s analysis model (dashed lines) to the scattered intensities. A model of drug–membrane interaction is proposed for each diffractogram together with the resultant chain packing.

The WAXS diffraction patterns for DPPC demonstrated an asymmetric double Bragg peak (d_20_ and d_11_ in Figure 3) that is characteristic of a pseudo-hexagonal tilted chain packing occurring in the L_β′_ phase.

The WAXS pattern of the addition of FTC to the L_β′_ phase showed a superposition of two chain packings. One resulted from a least perturbed lipid phase, where an intermediate tilt angle persists (peaks d_20_ and d_11_ and chain-packing scheme in Figure 3), and the other corresponded to a hexagonal chain packing resulting from the most influenced regions of the drug. Due to FTC’s hydrophilic nature, its insertion probably occurs at the headgroup region, causing a change in the area requirement of the headgroups, thus allowing the chains to be oriented in a hexagonal untilted chain packing (peak d_10_ and chain packing scheme in Figure 3). The insertion of FTC at the polar headgroup region could be further confirmed by the calculation of the lipids’ cross-sectional area (A_0_). An A_0_ of the 20.41 Å^2^/aliphatic chain was determined for DPPC, and was in good agreement with data previously reported [30]. The addition of FTC to DPPC reduced the A_0_ to the 20.15 Å^2^/aliphatic chain, ruling out the possibility of drug penetration within the aliphatic core of the membrane [48]. The addition of TDF to the L_β′_ phase promoted an approximation of the two Bragg peaks (peaks d_20_ and d_11_ and chain-packing scheme in Figure 3), suggesting that TDF is able to intercalate within the phospholipids and reduce their tilt angle. Upon the insertion of TDF, the lipid cross-sectional area suffers a slight increase (from 20.41 Å^2^ to 20.44 Å^2^). This indicates that the drug is also able to penetrate the aliphatic core of the membrane, being however well accommodated within the lipid chains, and thus not causing a great perturbing effect of the lipid packing. When both drugs are added to DPPC membranes, the effects observed were very similar to those of FTC addition.

**Figure 4:**
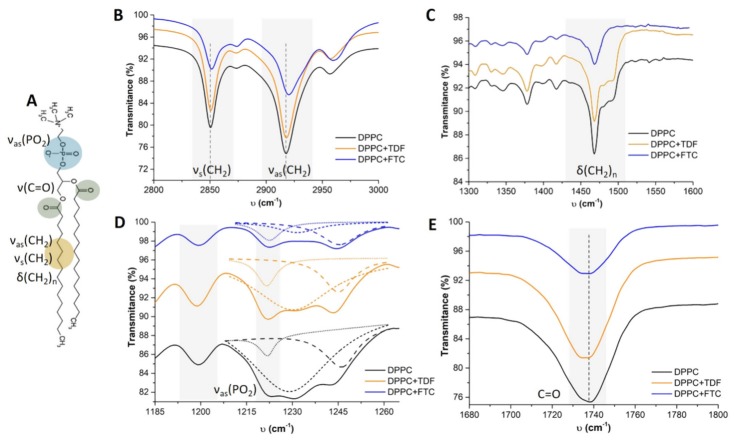
(**A**) DPPC phospholipid showing the chemical groups responsible for the main infrared vibration modes; (**B**–**E**) show attenuated total reflection–Fourier transform infrared (ATR–FTIR) spectra obtained for DPPC membranes in the absence (black) or in the presence of TDF (yellow) or FTC (blue) for different vibration modes: υ(CH_2_) stretching modes (**B**); δ(CH_2_)_n_ scissoring vibration mode (**C**); υ_as_(PO_2_) stretching mode (**D**); and υ(C=O) stretching mode (**E**). In panel (**D**), dashed lines represent the deconvolution of the bands performed to determine with precision the area of the band located at 1221 cm^−1^ required to subsequent calculation of the ratio a_1221_/a_1201_.

The impact of TDF and FTC in the microviscosity of DPPC membranes was further evaluated by ATR-FTIR. The results obtained by this technique may be confronted with SAXS and WAXS in the L_β′_ phase, since measurements were made at room temperature (below the *T*_m_ of DPPC). According to the vibrational frequencies obtained for the different chemical bonds of DPPC phospholipids (Figure 4A), multiple structural and biophysical information can be predicted upon interaction with drugs [49,50]: order of the phospholipid hydrocarbon tail assigned to υ(CH_2_) stretching modes with bands between 2800–3000 cm^−1^ (Figure 4B); lipid packing assigned to CH_2_ scissoring vibration mode (δ(CH_2_)_n_) with bands between 1300–1600 cm^−1^ (Figure 4C); hydrogen bonding or hydration capacity assigned both to phosphate headgroups (PO_2_) with bands between 1200–1260 cm^−1^ (Figure 4D); and to interfacial carbonyl groups (C=O) with a band close to 1736 cm^−1^ (Figure 4E). Calculation of the band areas ratio at 1221 and 1200 cm^−1^ (a_1221_/a_1201_), assigned to the ν_as_(PO_2_) stretching mode is also important to infer about the degree of hydration of the headgroup region. Increase of this ratio indicates dehydration or ion pair formation [50].

ATR-FTIR data indicates that FTC is able to induce higher effects in DPPC membranes, thus confirming the SAXS and WAXS results. It was possible to observe a shift of the CH_2_ symmetric (υ_s_(CH_2_)) and asymmetric (υ_as_(CH_2_)) stretching mode to higher frequencies (Figure 4B) in the presence of FTC, which is indicative of an increase of gauche rotamers accompanied with a more conformational disordered phase [51,52]. This confirms the hypothesis that FTC has a fluidizing effect in the lipid bilayer, thus justifying the reduction in the bilayer thickness observed in SAXS (Table 3). The CH_2_ scissoring vibrational mode of DPPC exhibits two characteristic peaks (Figure 4C) of an orthorhombic or pseudo-hexagonal tilted chain packing [49,50]. In agreement with WAXS data (Figure 3), FTC provoked the higher change in lipid packing, and the CH_2_ scissoring vibrational mode appeared as a singlet typical of a hexagonal lattice [49,50]. Regarding their position, the PO_2_ bands did not seem to be significantly affected by the drugs. However, analyzing the ratio of band areas (a_1221_/a_1201_) at the PO_2_ stretching modes (area of the bands at 1221 cm^−1^ and 1201 cm^−1^ in Figure 4D), it was possible to distinguish the effect of the drugs: a_1221_/a_1201_ of DPPC was 0.30, while the addition of TDF or FTC increased this ratio to 0.38 and 0.49, respectively. Such an increase indicated that dehydration or ion pair formation occurred [50], thus confirming the interaction of both drugs at the headgroup region that was already apparent from WAXS measurements. Furthermore, FTC, which is expected to interact mainly with the polar lipid headgroups, had the larger effect. The dehydration occurring in the L_β′_ phase observed by ATR-FTIR can explain the reduction in d values observed for both drugs in SAXS studies. On one hand, the drugs reduced the tilt and led to an increase in d values due to a more upright position of the acyl chains (Figure 3); on the other hand, the compounds induced a decrease in the water layer (as proved by an increase in a_1221_/a_1201_) that resulted in a decrease in the value of d. Both effects can compensate for each other, leading to a d value that, in the case of TDF, was closer to the d value of DPPC (Table 3). In the case of FTC, greater dehydration (as proved by an increase in a_1221_/a_1201_) together with a fluidizing effect (as proved by the shift of CH_2_ stretching modes to higher frequencies) can justify the accentuated d value decrease observed in SAXS (Table 3). Finally, the C=O stretching mode of DPPC at 1737 cm^−1^ was slightly shifted to lower frequencies (1735 cm^−1^) upon the addition of either FTC or TDF, which suggests that the drugs established hydrogen bonding to this group (Figure 4E).

Overall, ATR-FTIR and SAXS/WAXS measurements revealed that the interaction of FTC with DPPC bilayers promoted a decrease in bilayer thickness plus the water layer, and demonstrated to be highly disruptive of the lipid packing. Thus, despite being quite hydrophilic, FTC was able to interact with lipid polar headgroups, thus leading to dehydration and having a disruptive effect on lipid packing. This reinforces that FTC should not be considered for encapsulation in liposomes. Although TDF also interfered with lipid packing, its impact was smaller and more pronounced in DPPC gel phase (Figure 2A). Indeed, TDF moderate lipophilicity allowed for a better distribution of the drug within the lipid membranes, thus contributing to a minor impact in DPPC bilayers organization as compared to FTC. SAXS studies have also shown that fluid lipid systems may be less disturbed by TDF incorporation (Figure 2B) than ordered ones (Figure 2B). These conclusions further support the choice of liposomal formulations as adequate nanocarriers for TDF incorporation.

#### 3.1.3. Mucoadhesive Properties of Liposomes with Different Surface Charges

The diffusion of a drug nanocarrier through mucus depends not only on its diameter (should be up to around 500 nm) but mostly on the ability to establish adhesive interactions with mucins [53]. Electrostatic, hydrogen, and/or hydrophobic bonding can be established depending on the surface physicochemical properties of nanosystems. Although traditionally regarded as beneficial, mucoadhesion impairs the transport of nanomaterials in mucus. Thus, mucus-inert nanosystems have been considered as advantageous in order to allow better drug distribution and even retention in the vaginal cavity [54].

**Figure 5:**
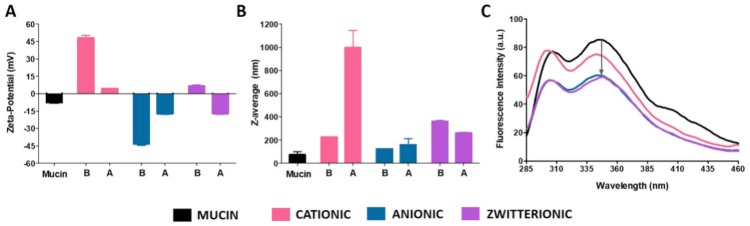
The effect of the surface charge of liposomes in zeta potential (**A**) and hydrodynamic diameter or Z-average (**B**), before (indicated by the letter B in *x*-axis) and after (indicated by the letter A in *x*-axis) interaction with a mucin suspension. Fluorescence emission spectra of mucin before interaction with liposomes and after interaction with cationic, anionic, or zwitterionic liposomes are presented in (**C**) (spectra follow the same color code used in bars of panels (**A**,**B**)).

Varying liposome composition may allow modulating their interaction with mucins. Therefore, we started by studying the interaction of cationic, zwitterionic, or anionic liposomes with mucin by assessing the hydrodynamic diameter (Z-average) and zeta potential of the lipid systems before and after incubation with mucin in suspension (Figure 5).

Cationic liposomes presented a significant decrease of zeta potential in the presence of mucin, down to values close to neutrality (Figure 5A), thus suggesting that interaction with mucin occurred mainly by adsorption to liposomes. Electrostatic interactions between positively charged amino quaternary groups of liposomes and negative sialic acid residues of mucin are likely involved. As a result, charge neutralization led to the formation of large aggregates, as evidenced by the increase in Z-average (Figure 5B). In the case of anionic liposomes, an increase in zeta potential was observed (Figure 5A) but without charge neutralization. This adds the possibility of the establishment of other type of interactions, namely of hydrophobic nature. Also, enduring negative surface charge was still sufficient to maintain liposomes in suspension due to the maintenance of electrostatic repulsion between vesicles (Figure 5B), as observed before for other type of negatively-charged nanosystems [55]. Finally, zwitterionic liposomes displaying in their headgroup region both positively (choline) and negatively (phosphate) charged groups observed a decrease in zeta potential from close to neutrality to negative values upon incubation with mucin (Figure 5A). In this case, positively charged choline likely interacted with the negatively charged sialic groups of mucin, leading to a prevailing of negatively charged phosphate groups at the surface of liposomes (Figure 5A). Also, the resulting net surface charge after incubation with mucin was able to avoid aggregation (Figure 5B).

We further complemented charge and size data by measuring the decrease in the intrinsic fluorescence of mucin upon incubation with the different type of liposomes. Close proximity or interaction between liposomes and fluorescent residues of mucin (namely tryptophan) are able to decrease overall fluorescence [56]. Fluorescence studies confirmed that all types of liposomes were able to interact with mucin (Figure 5C). Furthermore, it was possible to correlate the previous size and zeta-potential results with fluorescence quenching. Cationic liposomes interacted with mucin exclusively by electrostatic interactions, which probably occur at the surface without reaching tryptophan residues, and thus causing milder decrease in fluorescence. Quenching was more pronounced for anionic and zwitterionic liposomes, given that electrostatic interactions are possibly not the main mechanism of interaction with mucin, and hydrophobic interactions may account for the higher fluorescence quenching of tryptophan residues.

Overall, the data obtained indicate that positively charged liposomes are likely to exhibit less mobility in mucus by establishing strong electrostatic interactions with mucin. Conversely, low-affinity adhesive interactions with hydrophobic regions of anionic and zwitterionic liposomes may allow these last regions to feature enhanced transport in mucus, even if still featuring some degree of mucoadhesiveness. Our results are also in agreement with previous reports on the transport of differently charged nanosystems, including liposomes, in mucus or mucus surrogates [55,57].

Considering the putative advantages of having a nanocarrier that features, at least to some extent, enhanced transport across mucus, we discarded cationic liposomes for the encapsulation of TDF. The choice between negatively charged or zwitterionic liposomes, which displayed similar interaction with mucin, relied on previous reports that the zwitterionic nature of nanosystems (including several biological entities such as viruses) may further be advantageous when considering mucus transport [58]. Hence, zwitterionic liposomes were the only type considered for formulation studies.

### 3.2. Formulation Studies

#### 3.2.1. Physicochemical Characterization

Zwitterionic formulations with a common phosphatidylcholine (PC) headgroup and with different fatty acids (DMPC, DPPC, DSPC, and DOPC) were prepared without cholesterol and with 30% or 40% cholesterol (Table 1), and TDF was encapsulated in these formulations by different methodologies (hydration, incubation, and direct mixing, as represented in the Supplementary Materials, Figure S1). The main goal of testing different formulations was to assess how liposomes of different fluidities influence the encapsulation of TDF. The full results for the initial studies on EE% obtained for different methodologies of encapsulation and for different formulations are included in the Supplementary Materials (Figure S2). Values of EE% were generally high (74–89%), irrespective of the encapsulation method or the liposomal composition. All formulations/methodologies allowed producing liposomes with narrow sizes (134 ± 13 nm) and low PDI values (0.12 ± 0.06), thus suggesting the existence of monodisperse populations. The zeta-potential values of such liposomes varied between −10 and +10 mV, as expected for zwitterionic liposomes.

The evaluation of possible changes in the microviscosity of the liposomal formulation upon drug encapsulation was also required, as it is a helpful parameter to be considered in the choice of the most adequate formulation. For example, if including the drug in the liposomal formulation alters considerably its microviscosity, it can result in the liposomal system becoming too fluid to carry the drug during the required time after administration, and the lipid composition should perhaps be revised to convey more rigid domains [29]. Microviscosity changes induced by TDF encapsulation in lipid formulations of different fluidity (DMPC, DPPC, DSPC) were further evaluated by determining *T*_m_ (main phase transition temperature at which the liposomes undergo conformational changes from the gel phase to the fluid phase) and B (cooperativity of the phase transition, which provides evidences if the drug is homogeneously distributed). Results are included in the Supplementary Materials (Table S1). Overall, TDF encapsulation in different liposomes did not induce substantial changes to the microviscosity of lipid bilayers, thus indicating that none of the considered formulations were destabilized by drug incorporation.

In order to reduce the number of formulations being further evaluated, and since no formulation was shown to be particularly advantageous over the others, we decided to select only liposomes composed by DPPC. This lipid presents an intermediate fluidity and it also represents a prominent component of mucus phospholipids of cervicovaginal mucosa [59]. Also, pre-formulation studies were conducted by studying the interactions of TDF with liposomes composed by DPPC. The EE%, size, PDI, zeta-potential and biophysical parameters (*T*_m_, B) of TDF-loaded DPPC liposomes are summarized in Table 4.

All the different encapsulation methods demonstrated high and similar TDF EE% in DPPC liposomes correspondent to a total drug loading (ratio between the concentration of drug encapsulated and lipid concentration) of about 1%. The different preparation methods demonstrated similar mean diameters ranging between 113–130 nm. These values are considered suitable for allowing transport within the mucin mesh spacing in vaginal mucous fluids [60]. The PDI values were also low, regardless of the encapsulation method used and varied in the range of 0.06–0.24, thus indicating the presence of homogeneous and monodisperse liposome populations. Additionally, zeta-potential values within −10 and +10 mV confirmed the net neutral surface charge of the vesicles due to the balance of choline and phosphate residues present in the headgroup region of DPPC.

The experimental *T*_m_ value obtained (Table 4) for DPPC was in accordance with previous reported studies [47]. Additionally, neither the presence of TDF nor the encapsulation method used had a major impact in the *T*_m_ of DPPC, with a maximum variation of 0.44 °C observed upon the incorporation of TDF. The cooperativity of the phase transition of DPPC was high (2781 ± 234), indicating that the transition of lipid molecules to different phases occurred identically throughout the lipid bilayer, and at the same time, an uncommon increase in the cooperativity of the transition was observed in the presence of TDF. This may occur under circumstances where a compound penetrating the lipid bilayer has a planar non-bulky structure that is capable of parallel alignment to the phospholipids [61]. Thus, TDF not only did not disturb lipid packing, but also favored it by establishing van der Waals interactions with phospholipids. These results are in agreement with SAXS and WAXS results, which have shown that TDF is able to penetrate the aliphatic core of the membrane, but can still be well accommodated within the lipid chains, not affecting lipid packing.

Altogether, these results indicate that liposomes of DPPC are suitable for TDF encapsulation, since they feature: (i) high encapsulation efficiency (≈85%) for a total drug loading of ≈1%; (ii) adequate size and surface charge in order to allow at least partial transport across mucus; and (iii) structural stability, since liposomes are not fluidized by TDF incorporation, and their *T*_m_ is kept over physiological temperature.

#### 3.2.2. Pharmaceutical Performance of the Selected Formulation

Following the selection of TDF-loaded liposomes, we proceed with the in vitro evaluation of several parameters that are important predictors of the pharmaceutical performance. The proposed formulation, based in a hydrogel containing FTC and liposomes loaded with TDF, was tested for (i) drug permeation across a synthetic membrane simulating the epithelial barrier of the genital mucosa; (ii) the release profile of bioactive compounds; (iii) the rheological properties (which govern, at least partially, in vivo spreading and retention, as well as drug release behavior); and (iv) toxicity to relevant genital cell lines.

**Figure 6:**
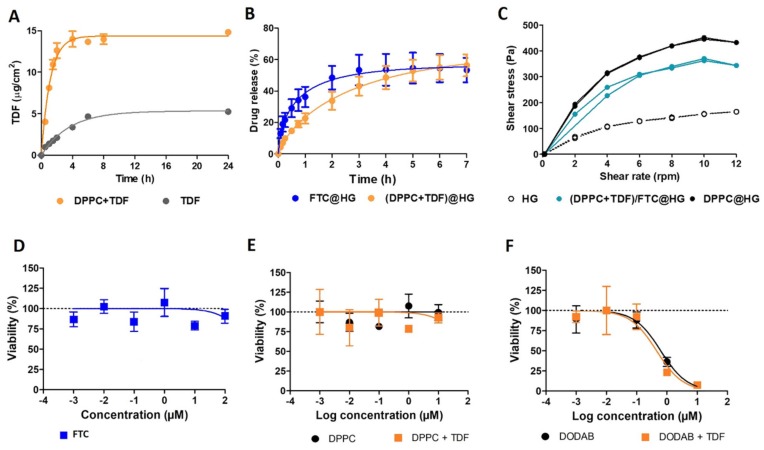
In vitro studies to evaluate the pharmaceutical performance of the developed formulation. In vitro TDF permeation across a polysulfone membrane in the free from (TDF) or when encapsulated in the DPPC liposomal formulation (TDF+DPPC) performed in aqueous medium at 37 °C (**A**). Release profile of TDF from DPPC liposomes included in hydrogels ((DPPC+TDF)@HG) and release profile of FTC from the hydrogels (FTC@HG) performed in aqueous medium at 37 °C (**B**). Flow rheograms of hydrogels without drugs or liposomes (HG), hydrogels with drug-free DPPC liposomes (DPPC@HG), and hydrogels containing FTC and TDF-loaded DPPC liposomes ((DPPC+TDF)/FTC@HG) (**C**). Viability of human HEC-1-A endometrial cell line was tested with increasing concentrations of FTC (**D**), liposomes of DPPC without drug (DPPC) or loaded with TDF (DPPC+TDF) (**E**), and liposomes of DODAB without drug (DODAB) or loaded with TDF (DODAB+TDF) (**F**). Concentrations in (**E**,**F**) are in TDF (real or virtual in the case of drug-free liposomes). Results are presented as mean ± standard deviation values (*n* = 3). Lines in (**A**,**B**) and (**D**–**F**) represent the Weibull model and log-logistic regression fits, respectively.

HIV-susceptible immune cells are mainly located at the sub-epithelial compartment of the cervicovaginal mucosa. TDF and FTC need to permeate the epithelium in order to reach this site and interact with such cells. The ability of FTC to penetrate the female genital tract is known to be greater than that of TDF, which has been related with the ability of the drug to inhibit its cell efflux [45]. Moreover, FTC is also able to interact with the lipid polar headgroups and lead to dehydration and a disruption of lipid packing, as shown in our drug–membrane interaction studies. This behavior may ultimately favor mucosal penetration through lipid bilayers impairment. In this scenario, it seems that an enhancement of membrane permeation may be particularly critical in the case of TDF, namely when encapsulated into liposomes. Comparison of the in vitro permeation profiles for unloaded free TDF or for TDF loaded in DPPC liposomes are shown in Figure 6A.

The flux of TDF in the free form across the membrane barrier was slower (*J*_ss_ = 0.717 ± 0.100 μg·cm^−2^·h^−1^), and diffusion started immediately (*t*_L_ = 0). Encapsulation in liposomes also allowed an immediate onset of TDF diffusion (*t*_L_ = 0), but with an accelerated flux rate across the membrane barrier (*J*_ss_ = 9.902 ± 0.542 μg·cm^−2^·h^−1^). Moreover, the liposomal formulation improved TDF permeation (*K_p_* = 0.979 ± 0·054 cm·h^−1^) as compared with the free drug (*K_p_* = 0.07129 ± 0.001 cm·h^−1^). One possible explanation for this distinct permeation behavior may be related with the large surface area of the liposomes permitting faster dispersion of the drug into the medium, thus increasing the concentration gradient of TDF at the membrane surface and increasing passive diffusion across this last. This enhancement in drug permeation seems interesting and indicative that liposomes may promote TDF concentration at the mucosal level. Still, any interpretation of these data needs to be cautious, since artificial membranes, such as the one used in our experiments, do not entirely mimic the complex permeation phenomena observed in vivo. Nevertheless, the value of synthetic membranes is well recognized for topical drug delivery, including by regulatory bodies such as the FDA [62]. Another aspect of our experiments has to deal with the lack of control of pH of the aqueous medium (≈5–6). Although deviating slightly from physiological values, TDF and FTC are neutral in a wide range of pH (as shown above during in silico studies), and thus hydronium concentration is unlikely to influence permeation in the considered system.

We next evaluated the release profiles of TDF from liposomes included in hydrogels and FTC from hydrogels (Figure 6B). The incorporation of the highly hydrophilic FTC in the semisolid pharmaceutical base allowed obtaining fast initial drug release, with 40% of the total FTC content being released in one hour. A release plateau was further reached at about 3 h, which lasted at least up to 7 h. These results indicate that high levels of FTC can be available for protection within a short timeframe after vaginal administration, while a residual drug can be released in a more sustained fashion. The release of TDF from the liposomal hydrogels was even slower and more sustained, with maximum drug levels being observed only after 5–6 h. Such behavior may be interesting, particularly due to the beneficial effects of liposomes in promoting permeation, as previously inferred.

The release profiles of both FTC and TDF were further adjusted to representative kinetic models, namely first-order, Korsmeyer–Peppas, Weibull, and Higuchi [33]. The calculated release kinetics parameters are included as Supplementary Materials (Tables S2–S5). The results of these analyses show that the best fits to the experimental data, with R^2^ values above 0.99, were obtained with the Weibull model. Accordingly, 56.5 ± 1.5% of FTC was released from hydrogels, and less time was required for the total release, indicating a higher rate of release (i.e., a parameter was higher), while 66.1 ± 2.5% of TDF was released from liposomal hydrogels at a slower rate of release (i.e., a parameter was lower) (Supplementary Materials, Table S4). Naturally, the need to bypass two barriers, namely the liposomal phospholipidic bilayer and the hydrogel matrix, may be responsible for the slower release rate of TDF as compared to FTC. Despite the valuable information provided by in vitro release assays, the intrinsic limitations of these studies should be taken into consideration, since several important events could influence drug release, particularly those occurring during sexual intercourse (e.g., increase in vaginal fluid volume, intense shear stress, presence of semen, etc.) [63].

The use of a hydrogel as a dosage form for TDF-loaded liposomes and FTC may help overcome vaginal retention issues and allow comfortable self-administration by women [64]. The pharmaceutical performance of hydrogels regarding in loco retention and spreading are well known to be partially determined by the viscoelastic properties of hydrogels, and thus, rheological characterization provides important data [65]. We obtained flow rheograms of hydrogels without drugs or liposomes (HG), liposomal hydrogels without the presence of drugs (HG+DPPC), and hydrogels containing FTC and TDF-loaded liposomes ((DPPC+TDF)/FTC@HG), as presented in Figure 6C. All hydrogels displayed a typical flow curve of a non-Newtonian pseudoplastic system, i.e., viscosity decreases with the increasing shear rate. This is in line with other vaginal gels available in the market and commonly used by women [65]. Such behavior may favor increased spreading during high shear rate events such as intravaginal administration and coitus. Low thixotropy (i.e., reduced hysteresis areas) indicates that, after the cessation of high shear stress, hydrogels can rapidly and almost fully resume their consistency and elastic features, thus promoting in loco retention. Still, some differences were noted among formulations. The incorporation of drug-free liposomes in the hydrogel caused a noticeable increase in viscosity. However, such increase was less pronounced in the presence of drugs. This seems to indicate positive and negative effects of liposomes and TDF/FTC, respectively, in the reinforcement of the polymeric matrix that forms the hydrogel. The onset of such effects requires more studies, but may likely be related with the electrostatic interactions between the components of the different hydrogels [66]. Similarly to our results, typical rheograms of non-Newtonian pseudoplastic behavior were also obtained by Pavelić et al. [67,68], Vanić et al. [69], and Malana et al. [70] for carbomer-based hydrogels, which displayed a continuous decrease of viscosity with the increase of shear rate. The rheological behavior of poly(acrylic acid) hydrogels was also evaluated by Ferreira et al. [71]. In agreement with our results, these researchers also demonstrated an increase in the gel viscosity upon the incorporation of liposomal formulations. Other studies failed to demonstrate significant differences in gel viscosity when compared with the control gels, while some of them report that the inclusion of liposomes promoted a decrease in this parameter. This was probably due to the presence of sodium ions in the buffer where the liposomes were prepared, which are not compatible with anionic carbomers [67,68].

Finally, we assessed the cytotoxic potential of free TDF and FTC, drug-free DPPC liposomes, and TDF- loaded liposomes using HEC-1-A and CaSki cell lines as in vitro models of the genital epithelium and relevance in the development of vaginal microbicides [72]. Hydrogels were not tested, since at relevant drug/liposome concentrations, the relative amount of formulation to cell culture medium was too high to be tested, resulting in excessive nutrient dilution and/or the high viscosity of the mix for cell culture experiments. Figure 6D–F presents the viability of HEC-1-A cells exposed to increasing concentrations of free FTC and different liposomes (with or without TDF). The half-maximal cytotoxic concentration (CC_50_) values calculated from log-logistic regressions for FTC were estimated to be above 100 μM (maximum tested concentration) for both considered cell lines (Figure 6D; data not shown for CaSki cells). These results are in accordance with previously reported studies [73]. In the case of free TDF, the toxicity potential was higher, with CC_50_ values of 34 μM and 55 μM being calculated for HEC-1-A and CaSki cells, respectively (Supplementary Materials, Figure S3). Again, these are in accordance with the reported values in the literature (22–50 μM), and indicate the higher cytotoxicity of TDF as compared to FTC [74,75]. When considering DPPC zwitterionic liposomes, concentrations above 10 μM in TDF could not be used due to the large amount of corresponding liposomes (drug loading was around 1%). Still, the viability profiles for both cell lines were similar up to at least the maximum TDF concentration considered, and thus, CC_50_ values could only be estimated to be well above 10 μM (Figure 6E; data not shown for CaSki cells). This suggests that the encapsulation of TDF in DPCC liposomes did not, at least, increase the intrinsic toxicity of the drug. To highlight the importance of the proper selection of liposomal composition, we also tested the cytotoxicity of cationic DODAB liposomes (Figure 6F). A typical reduction of around 2-log in CC_50_ values (0.4–1.3 μM) was observed for liposomes (either containing TDF or not) in both cell lines. Such exacerbated toxicity is likely associated with the cationic lipid component rather than TDF. Overall, the considered DPPC liposomes presented low cytotoxicity potential, with no apparent decrease in cell viability being observed at TDF concentrations that are at least around 200 times and 50 times higher than those required for the half-maximal inhibition of drug-susceptible or drug-resistant HIV-1 strains, respectively [74,75].

## 4. Discussion

The prevention of sexual transmission remains a top priority in the fight against HIV/AIDS. Despite all available and effective strategies, the number of infected people keeps on increasing. The development of topical PrEP approaches, namely by developing microbicides, holds great promise, but efficacy has been, at best, marginal. Novel formulation approaches are in demand. The present work attempts to propose a tentative topical product that could parallel the currently available oral PrEP with TDF/FTC. The rationale behind such approach relies on the premise that the ARVs used for PrEP act essentially at the mucosal level in order to block early HIV-1 transmission events. The strategy presented in this manuscript was heavily based on pre-formulation in silico, and experimental studies that highlighted the real ‘needs’ of the candidate drugs. Generated data guided the selection of an adequate formulation strategy (in particular for liposomal carriers) that could comply with physiological requirements pertaining to vaginal drug administration. We specifically proposed a novel topical microbicide based in liposomal hydrogels for the vaginal delivery of TDF/FTC. The composite system was further tested for important attributes that are able to determine its biopharmaceutical performance.

In silico pre-formulation studies were essential for predicting TDF/FTC physicochemical properties under relevant physiological conditions and guiding formulation/dosage form engineering. Both drugs presented limitations that dictated the need for suitable action. The moderate affinity to lipid environments and high solubility of TDF indicated that a hypothetical ideal system would have to contain both polar and nonpolar regions in order to allow drug solubilization. For this reason, we decided to proceed with the development of liposomes. In the case of FTC, its hydrophilicity guided our formulation efforts toward hydrogels.

SAXS, WAXS, and ATR-FTIR studies were also performed as part of the pre-formulation work. Important structural and biophysical information on the interaction of the drugs with lipid membranes were obtained, and helped us to better anticipate the behavior of TDF and FTC when reaching important barriers such as the ensemble cervicovaginal epithelial cell lining or individual cell membranes. SAXS and WAXS measurements supported TDF/FTC in silico predictions and confirmed our formulation approach. FTC was shown to have a significant impact in the structure of membranes: the drug caused a considerable reduction in the thickness of the bilayer, plus the hydration layer, which was probably due to a fluidizing action. A notorious disruptive effect in lipid packing was also observed in both gel and fluid lipid phases, which emphasizes the inadequacy of liposomes to encapsulate FTC. Conversely, lipid nanocarriers seem to be suitable for TDF, since this compound is distributed within the phospholipids of the lipid bilayers while causing a minor biophysical impact.

At this point, we decided that FTC should be incorporated into a hydrogel, while TDF should be loaded in a liposomal formulation. The next step comprised the selection of a suitable liposome composition for TDF encapsulation. Two essential parameters were studied, namely: (i) surface charge, which may be relevant for the interaction with mucus; and (ii) lipid fluidity, which is an important determinant of the overall physicochemical properties. Lipid fluidity was modified by changing the length of the lipid chains or by incorporating cholesterol. In vitro studies confirmed the strong electrostatic interaction between cationic liposomes and negatively charged mucin. Anionic and zwitterionic liposomes also established adhesive bonding with mucin, although of lower affinity and readily cleavable. Ultimately, zwitterionic liposomes based on DPPC appeared to be the better choice in order to assure a good balance between mucoadhesion and mucus diffusion.

TDF-loaded zwitterionic liposomes presenting sizes around 130 nm and narrow size distribution (PDI = 0.12 ± 0.06) were further prepared. The EE% was high (≈85%) and well above the values reported previously for TDF-loaded magneto-plasmonic DPPC liposomes [76]. However, in this last case, the higher drug-to-lipid ratios used, and the co-incorporation of magneto-plasmonic nanoparticles into liposomes may justify differences. Other types of nanocarriers have also been proposed for the encapsulation of TDF but lower—or, at best, similar—EE% values have been reported [77,78,79]. The biophysical stability of liposomes upon the incorporation of TDF was also evaluated, and the results supported that vesicles were not fluidized by drug incorporation and that their *T*_m_ was kept over the physiological temperature. Zwitterionic liposomes used for TDF encapsulation further demonstrated the ability to enhance the transport of TDF across a synthetic membrane barrier (*J*_ss_ = 9.902 ± 0.542 μg· cm^−^^2^·h^−1^) and significantly improved TDF permeation (*K_p_* = 0.979 ± 0.054 cm·h^−1^) as compared to the free drug (*K_p_* = 0.07129 ± 0.001 cm·h^−1^). Such observations were regarded as promising, and support that liposomes may be able to enhance TDF accumulation at mucosal tissues.

Drug release studies also yielded interesting indications regarding the ability of hydrogels and liposomes to release FTC and TDF, respectively. The ability to provide a considerable fraction of incorporated drugs in a relatively rapid fashion may allow achieving protective levels soon after vaginal administration. Also, slower release of the remaining drug content may allow sustaining FTC and TDF levels at the vagina for at least a few hours. Such a product could be particularly useful for the pericoital protection of women engaging in occasional sexual activity. The only comparable work focusing on a vaginal system for the co-delivery of TDF and FTC was just recently released, and considered drug-loaded Eudragit^®^ L 100 nanoparticles incorporated into soft polymeric films [77]. Although the rapid release of both drugs was also observed, no residual sustained release was noted, thus reflecting the difficulty of devising a simple and common vehicle that is able to provide biphasic release behavior for both TDF and FTC. The liposomal hydrogels were also shown to possess appropriate rheological behavior, namely when considering vaginal administration. The viscoelastic nature of the vehicle may be found to be useful in enhancing drug distribution and retention within the mucosal cavity, although advanced testing (namely in vivo) is deemed necessary to confirm these assumptions. Furthermore, the pH of the liposomal gel was slightly acidic (≈5.2) at the upper limit of the vaginal pH range considered as normal for healthy women of reproductive age. We used non-buffered aqueous medium as solvent for in vitro drug release studies, as neither the formulation nor the drugs are ionized in a wide range of pH values. Thus, pH changes are unlikely to influence the permeation/release or physicochemical characteristics in the considered system. Nevertheless, we plan on conducting future release studies in simulated vaginal fluid.

Finally, cytotoxicity studies demonstrated that no particular safety issues may be foreseen at this point, namely beyond those intrinsic to the incorporated drugs. Also, comparison between zwitterionic and cationic liposomes reinforces the choice of the previous when considering the development of microbicides. Safety is a cornerstone of the field, and even minor toxicity concerns at early stages of microbicide development are deemed unacceptable [80].

Overall, this research work represents, to the best of our knowledge, the first hydrogel-based formulation containing simultaneously FTC and TDF-loaded liposomes for vaginal administration and intended for topical PrEP. Although additional studies are required and envisioned—namely in vitro ARV activity, transport analysis of liposomes in the gel and mucus, ex vivo permeability, and in vivo pharmacokinetics, safety, and efficacy evaluation—the proposed formulation constitutes a potentially useful tool for advancing the microbicides field.

## Figures and Tables

**Table 1:** Lipid composition of the different liposomal formulations used throughout the work as well as the concentration and hydration and extrusion temperature used for their preparation. CHOL: cholesterol; DMPC: 1,2-dimyristoyl-sn-glycero-3-phosphocholine, DMPG: 1,2-dimyristoyl-sn-glycero-3-phosphorylglycerol, DODAB: dioctadecyldimethylammonium bromide, DOPC: 1,2-dioleoyl-sn-glycero-3-phosphocholine, DPPC: 1,2-dipalmitoyl-sn-glycero-3-phosphocholine, DSPC: 1,2-distearoyl-sn-glycero-3-phosphocholinephosphatidylcholine, TDF: tenofovir disoproxil fumarate.

Type of Studies	Lipid Composition	Concentration (mM)	Temperature Used in Hydration and Extrusion Process (°C)
Mucoadhesion studies	Zwitterionic liposomes:DMPC	0.4	40
	Anionic liposomes:DMPG		40
	Cationic liposomes:DODAB		60
Encapsulation of TDF (40 μM)	DOPC		
	DOPC:CHOL (7:3)	4	Room temperature
	DOPC:CHOL (6:4)		
	DMPC		
	DMPC:CHOL (7:3)	4	40
	DMPC:CHOL (6:4)		
	DPPC		
	DPPC:CHOL (7:3)	4	55
	DPPC:CHOL (6:4)		
	DSPC		
	DSPC:CHOL (7:3)	4	65
	DSPC:CHOL (6:4)		

**Table 2:** In silico prediction of several physicochemical descriptors obtained from TDF and FTC chemical structure using Chemaxon^®^ software.

Drug	MW (g·mol^−1^)	PSA (Å^2^)	VWSA (Å^2^)	logP	S (mg·mL^−1^)	pKa	H Donors	H Acceptors
TDF	635.52	185.44	753.35	2.65	1.24	3.75	1	10
FTC	247.24	88.15	279.83	-0.90	7.67	1.75	2	5

Abbreviations: logP—log_10_ of the octanol/water partition coefficient; S—intrinsic aqueous solubility; MW—molecular weight; pKa—negative log_10_ of the ionization constant; PSA—polar surface area; VWSA—Van der Waals surface area.

**Table 3:** Long spacings (d_L_) and correlation length (ξ) determined from SAXS patterns and short spacings (d_S_) determined from *p*-bromo benzoic acid (WAXS) patterns of DPPC without or with FTC, TDF, and FTC+TDF (3:2 *w*/*w*) in the L_β_′ and L_α_ phases.

Lipid Bilayers in the Absence or Presence of Drugs	Long-Spacing Values (SAXS) ^1^				Short-Spacing Values (WAXS)	
	d_L_^1^ (Å)		ξ		d_S_ (Å)	d_S_ (Å)
	L_β′_	L_α_	L_β′_	L_α_	L_β′_	L_β′_
DPPC	62.91	65.15	1084	1018	4.25	4.11
DPPC + FTC	51.80	54.21	41	24	4.21	4.11
DPPC + TDF	62.70	68.14	96	688	4.24	4.14
DPPC + FTC + TDF	53.36	53.55	41	42	4.19	4.11

^1^ The spacings were calculated for the first reflection order Bragg peak.

**Table 4:** Physicochemical and biophysical characterization of empty DPPC liposomes and DPPC liposomes loaded with TDF by different methods (incubation, hydration, and direct mixing), including: Encapsulation efficiency (EE%), mean hydrodynamic diameter (Z-average), polydispersity index (PDI), zeta potential, main phase transition temperature (T_m_), and cooperativity of the phase transition (B). R^2^ is the coefficient of determination of *T*_m_ and B after fitting data with Equation (1).

Type of Characterization	Parameters Evaluated	DPPC	DPPC+TDF		
			Incubation	Hydration	Direct Mixing
Physicochemical	EE (%)	-	84.63 ± 5.09 ^ns^	87.31 ± 4.68 ^ns^	84.79 ± 6.51 ^ns^
	Z-average (nm)	123.97 ± 3.62	113.13 ± 0.38 ^ns^	113.47 ± 1.07 ^**^	129.83 ± 6.69 ^**^
	PDI	0.11 ± 0.01	0.06 ± 0.01 ^**^	0.10 ± 0.01 ^***^	0.24 ± 0.01 ^***^
	Zeta potential (mV)	−3.55 ± 0.29	+6.00 ± 0.28 ^***^	+0.80 ± 0.33 ^***^	−5.29 ± 0.280 ^ns^
Biophysical	T_m_	41.05 ± 0.03	41.19 ± 0.05 ^ns^	41.27 ± 0.02 ^ns^	41.49 ± 0.03 ^ns^
	B	2781 ± 234	4117 ± 724 ^ns^	3922 ± 238 ^ns^	3135 ± 298 ^ns^
	R^2^	0.999	0.999	0.999	0.999

Results are the mean ± standard deviation of at least three independent measurements, and comparisons were performed using one-way ANOVA with the Tukey–Kramer post-test for the following paired observations: 1) EE% obtained with the incubation method vs. EE% obtained with the hydration method; EE% obtained with the hydration method vs. EE% obtained with the direct mixing method; EE% obtained with the direct mixing method vs. EE% obtained with the incubation method; 2) Z-average, PDI, zeta potential, T_m_, or B of: liposomes of DPPC loaded with TDF by the incubation method vs. liposomes of DPPC loaded with TDF by the hydration method; liposomes of DPPC loaded with TDF by the hydration method vs. liposomes of DPPC loaded with TDF by the direct mixing method; liposomes of DPPC loaded with TDF by the direct mixing method vs. liposomes of DPPC loaded with TDF by the incubation method; *** *p* < 0.001; ** *p* < 0.01; * *p* < 0.05; ns, not significant.

## Supplementary Materials

The following are available online at https://www.mdpi.com/1999-4923/11/9/485/s1, Figure S1. Schematic representation of encapsulation methods of TDF (final concentration 40 μM) in zwitterionic liposomes (4 mM) with different degrees of membrane rigidity; Figure S2. Encapsulation efficiency (EE%) of TDF (40 μM) in liposomes with different degrees of membrane rigidity (4 mM) by three encapsulation methods: hydration, direct mixing, and incubation. The dashed lines define the interval between the highest and lowest encapsulation efficiencies obtained; Table S1. Main phase transition temperature (*T*_m_) and cooperativity of the phase transition (B) of the lipid formulations DMPC, DPPC and DSPC (4 mM) before and after TDF (40 μM) encapsulation by three different methodologies (incubation, hydration, and direct mixing); Tables S2–S5. Fitting of FTC release from hydrogel (HG) and TDF release from liposomes included in hydrogel (LH) in aqueous medium (37 °C); Figure S3. Viability of HEC-1-A and CaSki cells with increasing concentrations of TDF. Results are presented as mean ± standard deviation values (*n* = 3). Lines represent log-logistic regression fits.
