# Supplementary material for: Rational Development of Liposomal Hydrogels: A Strategy for Topical Vaginal Antiretroviral Drug Delivery in the Context of HIV Prevention
Source: Pharmaceutics. 2019 Sep 18;11(9):485. doi: 10.3390/pharmaceutics11090485 (PMC6781289; doi:10.3390/pharmaceutics11090485)
Supplement: Supplementary file 1 [file pharmaceutics-11-00485-s001.pdf]

pharmaceutics

Supplementary Materials: Rational Development of Liposomal Hydrogels: A Strategy for Topical Vaginal Antiretroviral Drug Delivery in the Context of HIV Prevention

Maria J. Faria, Raul Machado, Artur Ribeiro, Hugo Gonçalves, Maria Elisabete C. D. Real Oliveira, Teresa Viseu, José das Neves and Marlene Lúcio

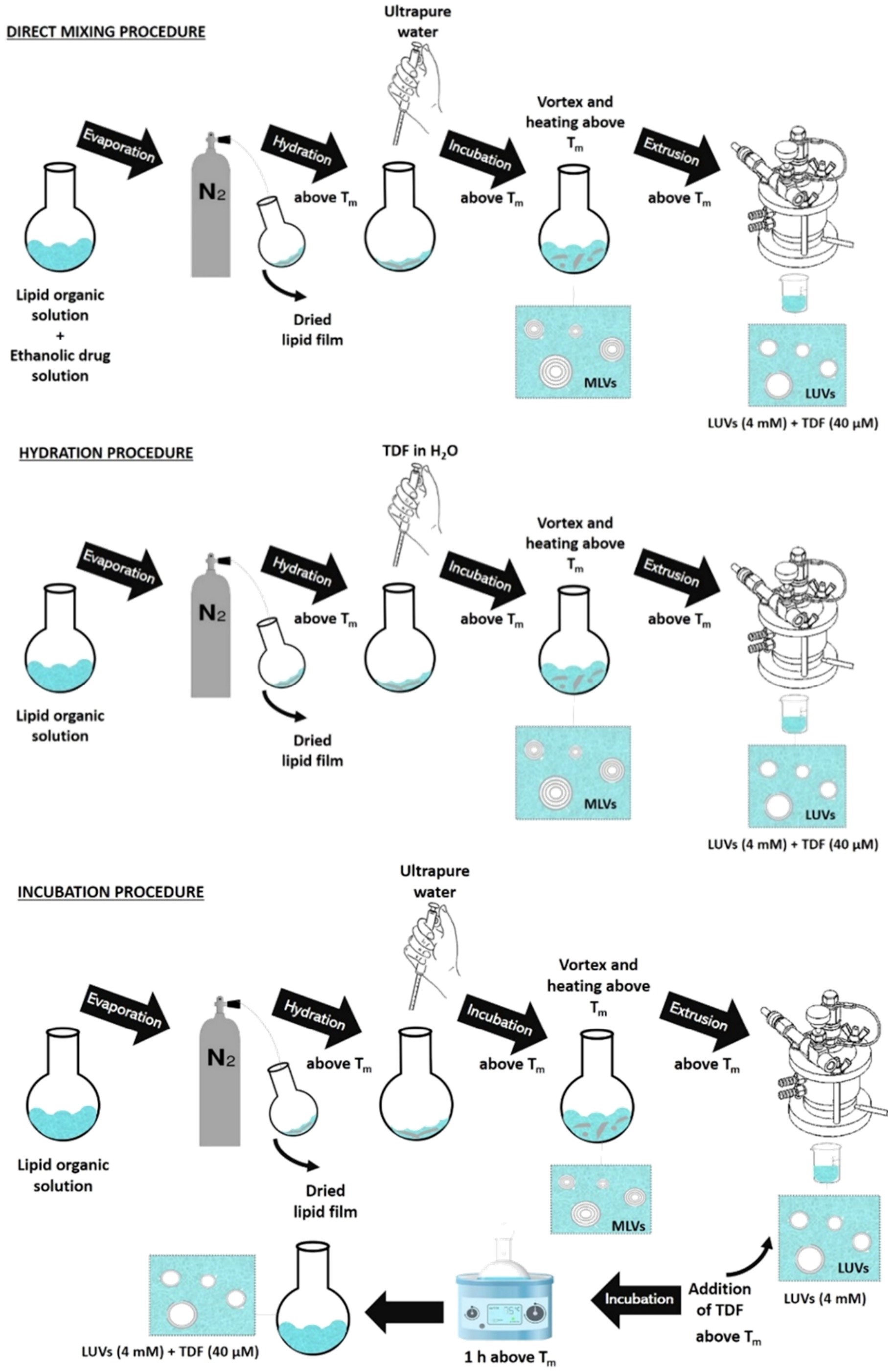

Figure S1. Schematic representation of encapsulation methods of TDF (final concentration 40 µM) in zwitterionic liposomes (4 mM) with different degrees of membrane rigidity.

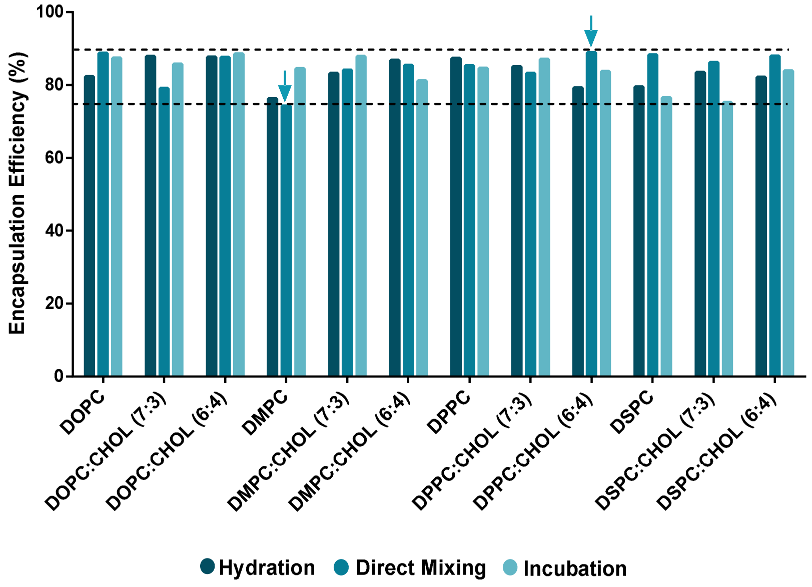

Figure S2. Encapsulation efficiency (EE %) of TDF (40 µM) in liposomes with different degrees of membrane rigidity (4 mM) by three encapsulation methods: hydration, direct mixing and incubation. The dashed lines define the interval between the highest and lowest encapsulation efficiencies obtained.

Table S1. Main phase transition temperature (T_m) and cooperativity of the phase transition (B) of the lipid formulations DMPC, DPPC and DSPC (4 mM) before and after TDF (40 µM) encapsulation by three different methodologies (incubation, hydration and direct mixing).

	DMPC	DMPC+TDF		
		Incubation	Hydration	Direct Mixing
T_m	23.76 ± 0.023	23.96 ± 0.02	23.79 ± 0.15	22.28 ± 0.41
B	857.70 ± 37.56	695.12 ± 34.29	514.99 ± 146.57	237.35 ± 41.24
R^2	0.999	0.999	0.848	0.991
	**DPPC**	**DPPC+TDF**		
		**Incubation**	**Hydration**	**Direct Mixing**
T_m	41.05 ± 0.03	41.19 ± 0.05	41.27 ± 0.02	41.49 ± 0.03
B	2780.89 ± 234.32	4116.88 ± 724.16	3921.77 ± 237.81	3135.08 ± 297.54
R^2	0.999	0.999	0.999	0.999
	**DSPC**	**DSPC+TDF**		
		**Incubation**	**Hydration**	**Direct Mixing**
T_m	54.08 ± 0.02	54.198 ± 0.07	54.45 ± 0.02	54.07 ± 0.02
B	4400.78 ± 391.19	3302.02 ± 556.45	6237.21 ± 668.93	4912.77 ± 290.35
R^2	0.999	0.975	0.997	0.989

Table S2 to S5. Fitting of FTC release from hydrogel (HG) and TDF release from liposomes included in hydrogel (LH) in aqueous medium (37 °C).

Table S2. 1st Order kinetics.

Mathematical Equation	pH	Adjusted Parameters	R^2	R^2 Adjusted
$F_{max}(1-e^{-kt})$	HG with FTC	F_{max} = 52.97 ± 1.628<br>k = 1.597 ±0.1882	0.9620	0.9585
	LH with TDF	F_{max} = 57.14 ± 1.461<br>k = 0.5026 ± 0.035	0.9934	0.9928

F_{max} is the total amount of drug released and k is the first order release constant.

Table S3. Korsmeyer–Peppas Model.

Mathematical Equation	pH	Adjusted Parameters	R^2	R^2 Adjusted
at^n	HG with FTC	a = 35.17 ± 1.322	0.9598	0.9561
		n = 0.2728 ± 0.02555		
	LH with TDF	a = 22.72 ± 0.9262	0.9875	0.9875
		n = 0.5049 ± 0.02603		

a is a constant of geometric and structural incorporation that takes into account the pharmaceutical form; n is a release representing the mechanism of diffusion of the drug, being based on Fick's law, (a value of n equal to or less than 0.5 indicates a Fickian diffusion, for values between 0.5 and 1 indicates a non- Fickian diffusion).

Table S4. Weibull Model.

Mathematical Equation	pH	Adjusted Parameters	R^2	R^2 Adjusted
$(1 - e^{(-at^b)})$	HG with FTC	a = 1.159 ± 0.08798	0.9945	0.9934
		F_{max} = 56.49 ± 1.451		
		b = 0.6314 ± 0.04353		
	LH with TDF	a = 0.7885 ± 0.02721	0.9990	0.9988
		F_{max} = 66.11 ± 2.494		
		b = 0.4343 ± 0.02335		

F_{max} is the total amount of drug released and a is a parameter a that defines the time scale. The parameter b characterizes the shape of the curve. For (b = 1) (Case 1), sigmoid, for (b > 1) (Case 2) S-shaped, curvature upwards followed by a turning point, for parabolic (b < 1) (Case 3) with greater initial slope and then exponential.

Table S5. - Higuchi Model.

Mathematical Equation	pH	Adjusted Parameters	R^2	R^2 Adjusted
$kt^{0,5}$	HG with FTC	k = 26.02± 1.735	0.7402	0.7402
	LH with TDF	k = 22.87± 0.4112	0.9885	0.9885

K is the Higuchi dissolution constant.

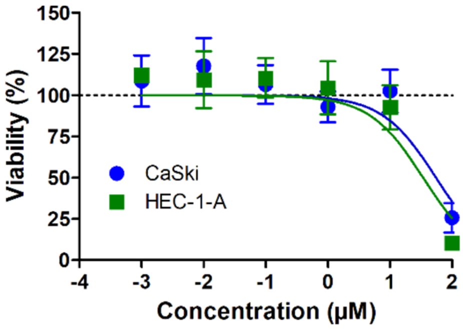

Figure S3. Viability of HEC-1-A and CaSki cells with increasing concentrations of TDF. Results are presented as mean ± standard deviation values (n = 3). Lines represent log-logistic regression fits.

© 2019 by the authors. Submitted for possible open access publication under the terms and conditions of the Creative Commons Attribution (CC BY) license (http://creativecommons.org/licenses/by/4.0/).
